# A comparative study on the leaf anatomical structure of *Camellia oleifera* in a low-hot valley area in Guizhou Province, China

**DOI:** 10.1371/journal.pone.0262509

**Published:** 2022-01-20

**Authors:** Yang Hu, Lu Yang, Chao Gao, Desheng Liao, Li Long, Jie Qiu, Hongli Wei, Quanen Deng, Yunchao Zhou

**Affiliations:** 1 Institute for Forest Resources and Environment of Guizhou, Key laboratory of forest cultivation in plateau mountain of Guizhou province, College of Forestry, Guizhou University, Guiyang, China; 2 Guizhou Southwest Karst Regional Development Institute, Xingyi, China; Shandong Normal University, CHINA

## Abstract

The leaf serves as an important assimilation organ of plants, and the anatomical structure of leaves can reflect the adaptability of the plant to the environment to a certain extent. The current study aimed to cultivate superior local cultivars, and 35 healthy individual plants were selected from the *Camellia oleifera* germplasm resource nursery for a comparative study of the leaf structure. In July 2019, the leaves were collected from 35 selected healthy *C*. *oleifera* plants, and the leaf structure was observed by using the paraffin section method. Healthy individual plants were screened using variance analysis, correlation analysis and cluster analysis. The representative indices were selected according to the cluster membership, correlation indices and coefficient of variation (C/V) for a comprehensive evaluation of drought resistance via the membership function. There were extremely significant differences in 11 indices of leaf structure for these 35 healthy plants. C18 had the greatest leaf thickness, C7 the largest spongy tissue, and C38 the largest ratio of palisade tissue thickness to spongy tissue thickness (P/S). The clustering results of the healthy individual plants differed significantly. The membership function showed that the drought resistance of 35 *C*. *oleifera* plants was divided into five categories. C18 had very strong drought resistance, and C3, C7 and C40 had strong drought resistance. There were significant differences in terms of the upper epidermis, P/S ratio and spongy tissue among the *C*. *oleifera* plants. C18, C3, C7 and C40 exhibited satisfactory drought resistance. Although C39 and C26 had moderate drought resistance, their P/S ratios were high, which might be used to cultivate high-yield and drought-resistant *C*. *oleifera* varieties. The leaf P/S ratio of *C*. *oleifera* from low-hot valley areas was high. Among various leaf structures, spongy tissue, upper epidermis, P/S ratio and cuticle constitute the drought resistance evaluation indices for *C*. *oleifera* grown in low-hot valley areas.

## Introduction

The leaf is an assimilation organ of the plant whose photosynthesis involved in its structural response to drought stress is complex, involves interactions between different structural levels [1,2], and directly affects photosynthetic factors such as water retention in the leaf, CO_2_ stomatal conductance, and mesophyll conductance [3,4]. Water and CO_2_ are substrates for photosynthesis. Under drought conditions, stomatal closure is an early adaptation of plants to cope with water deficit but also limits CO_2_ uptake by leaves [5]. At this time, how other tissue structures maintain photosynthesis under drought conditions is of crucial importance for plants [6].

Under drought stress, to maintain its biological function to ensure the normal operation of photosynthesis, the leaf is often required to change its anatomical structure to adapt to the environment. Therefore, the same species may evolve different structural characteristics when faced with varying degrees of drought stress. There are differences among individuals within the population. Additionally, facing the same degree of drought stress, the same species may also exhibit different structural characteristics [7,8], which is also referred to as leaf plasticity; the plasticity of leaf anatomical structure and physiology is an important guarantee for plants to adapt to adverse environment [9]. Among the various anatomical characteristics, the ratio between palisade tissue and spongy tissue (P/S) is positively correlated with the net photosynthetic rate in a significant (r = 0.9) or a very significant (r = 0.985) manner [10,53]. Leaf anatomical structure reflects important photosynthetic physiological characteristics and is closely associated with function, and therefore, leaf structure is widely used to evaluate the drought resistance of different varieties of the same species [11–13].

Guizhou Province is the center of karst topography in Southwest China. The soil in karst areas is rich in calcium and characterized by severe erosion. The direct leakage of water content in soil underground is the main form of soil erosion. The growth and development of plants in karst areas are often accompanied by environmental conditions featuring water deficits and high calcium [14–17]. In Guizhou Province, soil erosion is mainly distributed in Wangmo County and Ceheng County in Southwest Guizhou [18]. In addition, due to gradual reductions in annual precipitation in recent years [19], droughts occur frequently as a result. The Beipan River flowing through Guizhou Province forms a low-hot valley area, where a distribution of *Camellia oleifera* is found. However, related research has not been conducted in this area. However, the seeds of *Quercus sichourensis* distributed in this area show higher drought resistance than those in other distribution areas [20]. In Hunan Province, the distribution center of *Camellia oleifera*, the annual precipitation is approximately 1450 mm, whereas that in low-hot valley areas is approximately 1200 mm; in the meantime, for karst topography in low-hot valley areas, water loss is more likely to occur. Therefore, *C*. *oleifera* in the low-hot valley area may have the potential for undeveloped germplasm resources with desirable drought resistance.

*Camellia oleifera* is widely distributed in southern China, but not all distribution areas can be called the most suitable cultivation area. One of the possible reasons for this is that *C*. *oleifera* is a calciphobous plant [21]. Therefore, the National Forestry and Grassland Administration issued a development plan for the *C*. *oleifera* industry in China and classified areas suitable for cultivation of *C*. *oleifera* according to its distribution areas into the most suitable cultivation area, suitable cultivation area and relatively suitable area (arranged in descending order). The habitat of the last two areas is worse than that of the most suitable one. As a result, *C*. *oleifera* distributed in the last two areas is prone to more abiotic stress, which also makes the local *C*. *oleifera* cultivars better adapted to these distribution areas. Therefore, it is one of our tasks to select local *C*. *oleifera* cultivars [22]. For this purpose, we selected 35 healthy individual plants in this study (to be published) as materials for seed breeding according to the yield criterion.

At present, the seed breeding of *C*. *oleifera* in the low-hot valley area is mainly to select healthy individual plants. However, currently, the adaptative mechanism of *C*. *oleifera* in the low-hot valley area is still poorly understood because the leaf structure of the plant reflects the plant’s adaptation to the environment to a certain extent. Therefore, we conducted a comparative study to investigate the leaf anatomical structure of individual *Camellia oleifera* plants grown in the low-hot valley area in Guizhou Province, China. The following issues would be clarified by studying the leaf structure of *C*. *oleifera*. (1) What are the characteristics of anatomical structure of *C*. *oleifera* leaves in the low-hot valley area, and are there any differences between the anatomical structure of its leaves and those in other cultivation areas reported in the literature? (2) How does the leaf structure of *C*. *oleifera* in the low-hot valley adapt to water deficit and high calcium? (3) Are there any differences in drought resistance in terms of leaf anatomical structure for the 35 selected healthy *C*. *oleifera* plants? Our research is helpful in explaining how the leaf structure of *C*. *oleifera*, a calciphobous plant, adapts to drought and high-calcium environments in karst areas and will provide a theoretical basis for the selection of *C*. *oleifera* breeding materials in the low-hot valley area.

## Materials and methods

### Sample collection

**Fig 1:**
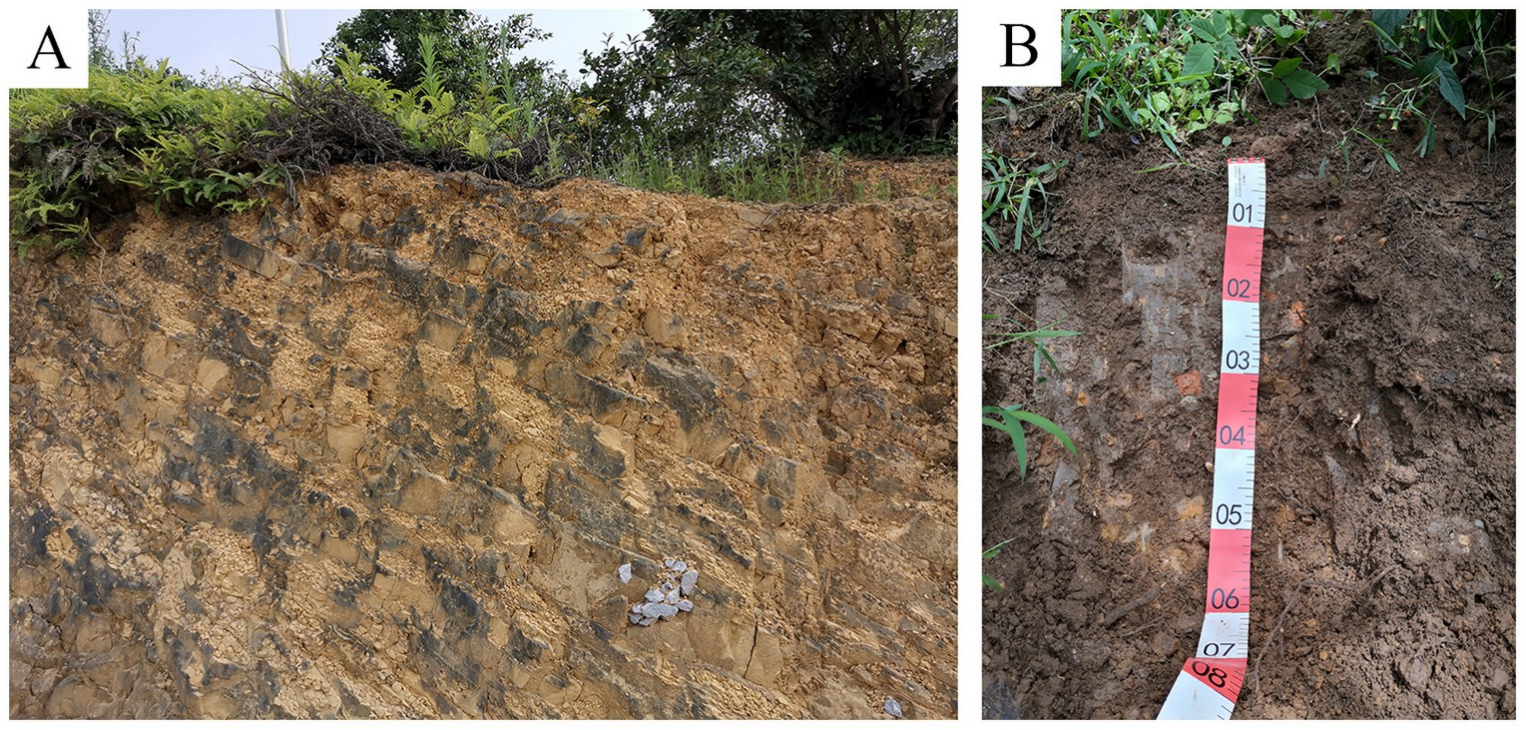
Soil profile in the *C*. *oleifera* study area and at the resource nursery. A. Karst topography. B. The soil layer of the nursery.

Leaf samples of healthy individual plants were collected in 2019 at the Guizhou University *Camellia Oleifera* Research Station, located in Ceheng County in southwestern Guizhou Province, China (24.71°-24.94° N, 105.79°-106.05° E), which has an average annual temperature of 19.7 °C, an average temperature of 27.2 °C in the hottest month and 10.5 °C in the coldest month, an annual accumulation temperature >10°C of 6,348 °C, an annual precipitation of 1,197 mm, heat and precipitation in the same seasons, precipitation from November to April accounting for only 16% of the annual precipitation, a tendency of rising temperature, little rainfall and frequent droughts in March and April, a rainy season starting from May, and an annual sun exposure of 1,257 h. Biyou Town and Yangba Town are the two primary distribution areas of *C*. *Oleifera* in Ceheng County. At these two towns, the planting area reached up to 5786.67 hm^2^, which accounted for 53.90% of that of the whole county. These two towns are also the main excellent *C*. *Oleifera* seed supply sources in the low-hot valley area in Guizhou Province. In this study, the study area was mainly centered on Biyou Town. In the study area, the soil was the typical yellow soil in karst regions of Southwest China. Severe soil erosion leads to a shallow soil layer, and the resulting drought problem is a great challenge faced by *C*. *oleifera* planting in this area (Fig 1A).

The nursery was established as follows. In the study area, *C*. *oleifera* plants with satisfactory economic characteristics were recorded (S2 Table). Their seeds were collected and then sowed in 2007 and 2008. In the sowing year, the land was prepared before sowing. To guarantee no less than one plant from the same preliminarily selected individual survived to blossom and bear fruit (normally eight years), three repetitions were set. The planting density was 2000 plants per hectare, which was slightly higher than the recommended density in the literature (900–1,800 plants per hectare) [23]. Due to differences in individual development, the *C*. *oleifera* plants also differed somewhat in canopy height, which led to inconsistent sunlight exposure among them or, even worse, disadvantageous tree death during the long-term photosynthesis-biomass accumulation process [24]. This phenomenon indicates that differences in the competitiveness to sunlight exist among the individuals.

At the time of sample collection, the trees aged 12–13 years, and they all reached fruit productive age [23]. The *C*. *oleifera* resource nursery is on the south slope. The soil is yellow soil with the soil layer thickness of 51.6±8.2 cm (Fig 1B). Between 2013 and 2018, the economic characteristics of the individual plants at the nursery were consecutively determined, and the plants growing at the stand edge were excluded. Within the involved six years, yield was observed within the late three continuous years. All these individuals were planted within two years, and their flowering and fruiting time had a difference of 1–2 years. This long-term determination was mainly based on the considerations that the growth differences among the trees led to the differences in the flowering and fruit setting periods and that long-term observation could help obtain more stable information about the trees.

We selected 45 healthy *Camellia oleifera* plants in total. The individual plants were named (C-number) according to the sequence of the healthy individual plants identified. Their yields per unit crown width were recorded (S1 Table).

For the current purpose, we excluded individuals with unstable yields and/or severe insect and pest diseases (original number was used). Insect and pest diseases mainly occurred between 2010 and 2011. The primary reason was that drought persisted in Southwest China in 2019 and 2010 [25], which weakened the resistance of young *C*. *oleifera* trees in Ceheng county [26]. Moreover, the high temperature accompanied by drought might bring the first appearance and peak of pests to earlier dates, which had adverse effects on *C*. *oleifera* [27]. Additionally, *C*. *oleifera* is plant of flowering-fruiting synchronization, and it normally takes 18–19 months from bud differentiation to fruit maturation. The number of differentiated flower buds in one year (it is the basis of fruit yield in the second year) is negatively correlated with fruit development in the same year, which leads to instable yields in successive years [22]. Although instable yields can be remedied though additional cultivation measures, such as artificial flower and fruit thinning and water and fertilizer management, these measures increase the production cost. Finally, 35 healthy plants were selected from the resource nursery for a comparative study of leaf structure.

There is an overlapping period between flower bud differentiation and fruit growth of *C*. *oleifera*, particularly in July and August each year. This period is the key time of oil conversion in *C*. *oleifera* fruit as well as the time of the *C*. *oleifera* leaf with the highest photosynthetic efficiency; the photosynthetic products of the leaves close to the base of the current-year branch are mainly provided for fruit development, whereas those with the top leaves are mainly provided for flower buds [28]. Considering that the competitiveness of the excellent plants differed somewhat, the leaves directly exposed to sunlight were taken as the samples. Therefore, in July 2019, three branches in the upper and middle parts of the east-facing canopy were selected from 35 healthy plants, and three leaves, the 5th to 7th healthy and mature leaves in the *C*. *oleifera* phyllotaxis, were selected from each branch. A total of nine leaf replicates were selected from each plant. Then, 0.5*0.5 mm leaf tissue was removed from the middle of each leaf while avoiding the midrib, which was immediately placed into carnoys fixative (95% ethanol: glacial acetic acid (V/V) = 3:1) for a fixation of 6 h. The leaves of the same plant were fixed in the same bottle. The bottle was exhausted of air after the fixation was completed, transferred to 70% alcohol solution and stored in a 4 °C refrigerator.

### Precipitation in the study area

The percentage of precipitation anomaly (Pa) can visually reflect the drought condition confronted by plants in specific areas, and Pa is calculated as follows [29]:

$$Pa=(P-\bar{P})/\bar{P}$$

where $\bar{P}$ represents the average monthly precipitation and *P* is the precipitation in a specific month. Pa > 0.4 indicates drought stress. The data used for precipitation analysis in this study were from the China Meteorological Information Center (http://data.cma.cn/).

### Observation of leaf anatomical structure

The fixed material was dehydrated in ethanol, made transparent in xylene, embedded in paraffin, and then sliced using a microtome (Leica RM 2235, Germany) with a section thickness of 8 μm. Safranine O-fast green staining was performed [30] after the specimen was dried. Then, it was sealed using neutral resin and photographed with an optical microscope (Leica DM 3000, Germany). Data were measured by ImageJ (avoiding leaf veins and heterolayer epidermis). Three measurements were performed for each leaf, and an average was obtained. The measured parameters included leaf thickness (LT), cuticle thickness, thickness of upper epidermis (TU), thickness of palisade tissue (TP), thickness of spongy tissue (TS), thickness of lower epidermis (TL), and thickness of first layer palisade tissue (TFP). The organizational tightness (CTR), tissue porosity (SR), upper and lower epidermis thickness ratio (U/L), and the P/S ratio were calculated. CTR = CTR = TP/LT * 100; SR = SR = TS/LT * 100; U/L ratio = upper epidermis thickness to lower epidermis thickness; P/S ratio = TP/ TS. Coefficient of variation (C/V) = standard deviation/mean ×100%, the plasticity index (PI) of leaf tissue structures = the minimum mean of an index of an individual plant/the maximum mean of the index of the individual plant [31].

### Data processing

Excel 2010 was used to sort out measurement results, Photoshop cs6 for plotting, SPSS19.0 for variance analysis, and Duncan for multiple comparison and hierarchical clustering of the data. The leaf tissue structure of an individual plant is indicated by the mean±SD. The rest is described by the mean. The mean leaf tissue of an individual plant was selected for clustering analysis.

According to the clustering of organizational structure, the typical indices were calculated by the correlation index [32], whose formula is:

$$R_{i}^{2}=\sum r^{2}/\left(n-1\right)$$

where $R_{i}^{2}$ is the correlation index for each index in each category, n is the number of indices in each category, and r is the correlation coefficient between one index and other indices in the same category. When there is only one index in the same category, the value of the correlation index is 1. A larger correlation index between a certain index and other indices in the same category better represents the characteristics of this category. With reference to the correlation index and C/V for index selection, the drought resistance of leaves of 35 *C*. *oleifera* plants was evaluated in terms of the leaf anatomical structure by using the membership function in fuzzy mathematics [33]:

$$f\left(x_{i}\right)=\left(x_{i}-x_{min}\right)/\left(x_{max}-x_{max}\right)$$

where *f*(*x*_*i*_) is the value of the drought resistance membership function; *x*_*i*_ is the mean leaf tissue structure of individual plants; and *x*_*min*_ and *x*_*max*_ are the average minimum and maximum of the values measured for the leaf tissue structure of all individual plants, respectively. If a certain index is negatively correlated with drought resistance, then the inverse membership function is used for conversion. The calculation formula is:

$$f\left(x_{i}\right)=1-\left(x_{i}-x_{min}\right)/\left(x_{max}-x_{min}\right)$$

After calculating the average membership degree of the indices, the healthy individual plants with a higher average degree of membership have a better stress resistance.

## Results

### Precipitation at the leaf development stage of *C*. *oleifera* in Ceheng in 2019

**Fig 2:**
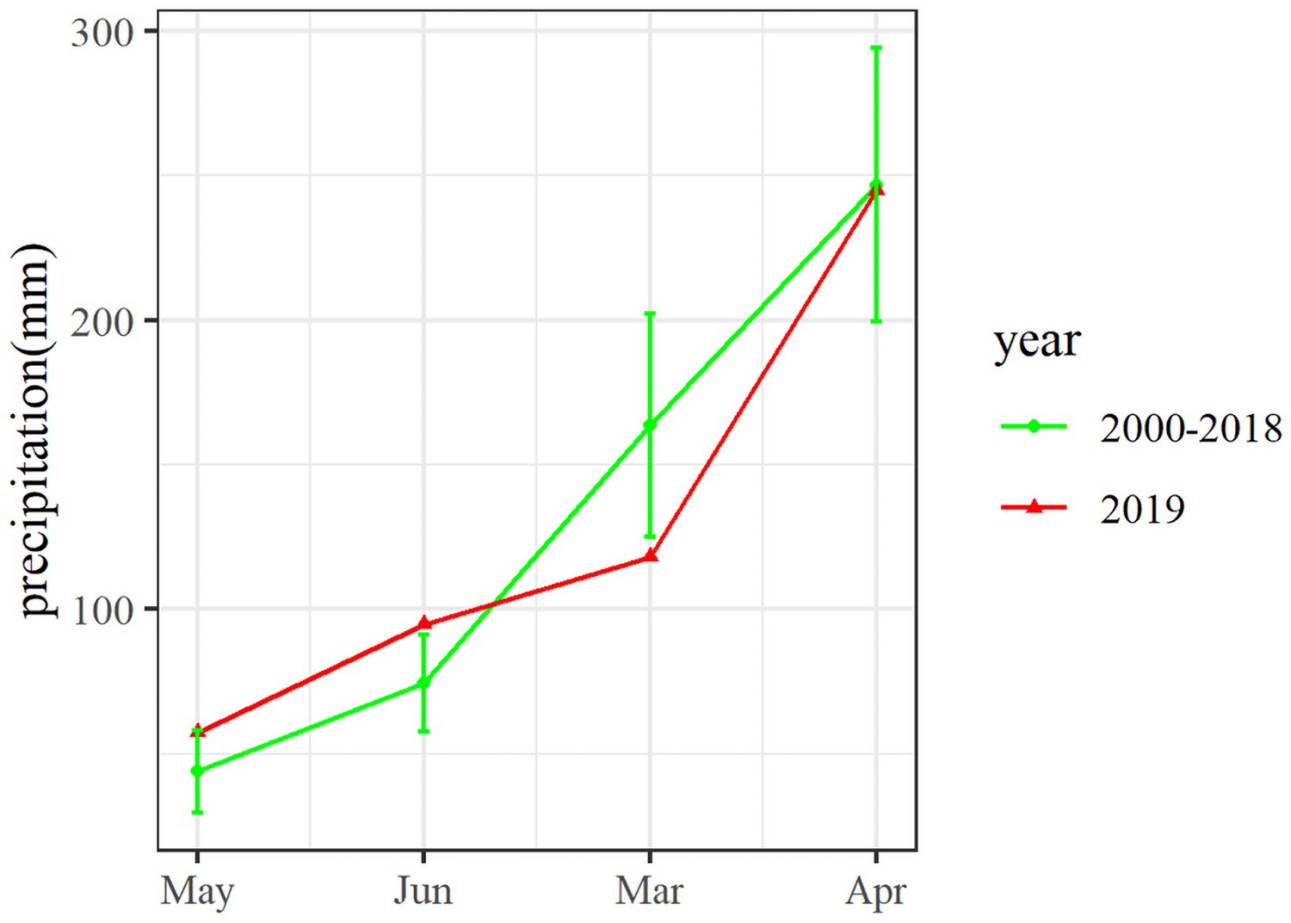
Precipitation between March and June in 2000–2018 and that in 2019. The fold line is the average and the green vertical line is the standard deviation.

**Fig 3:**
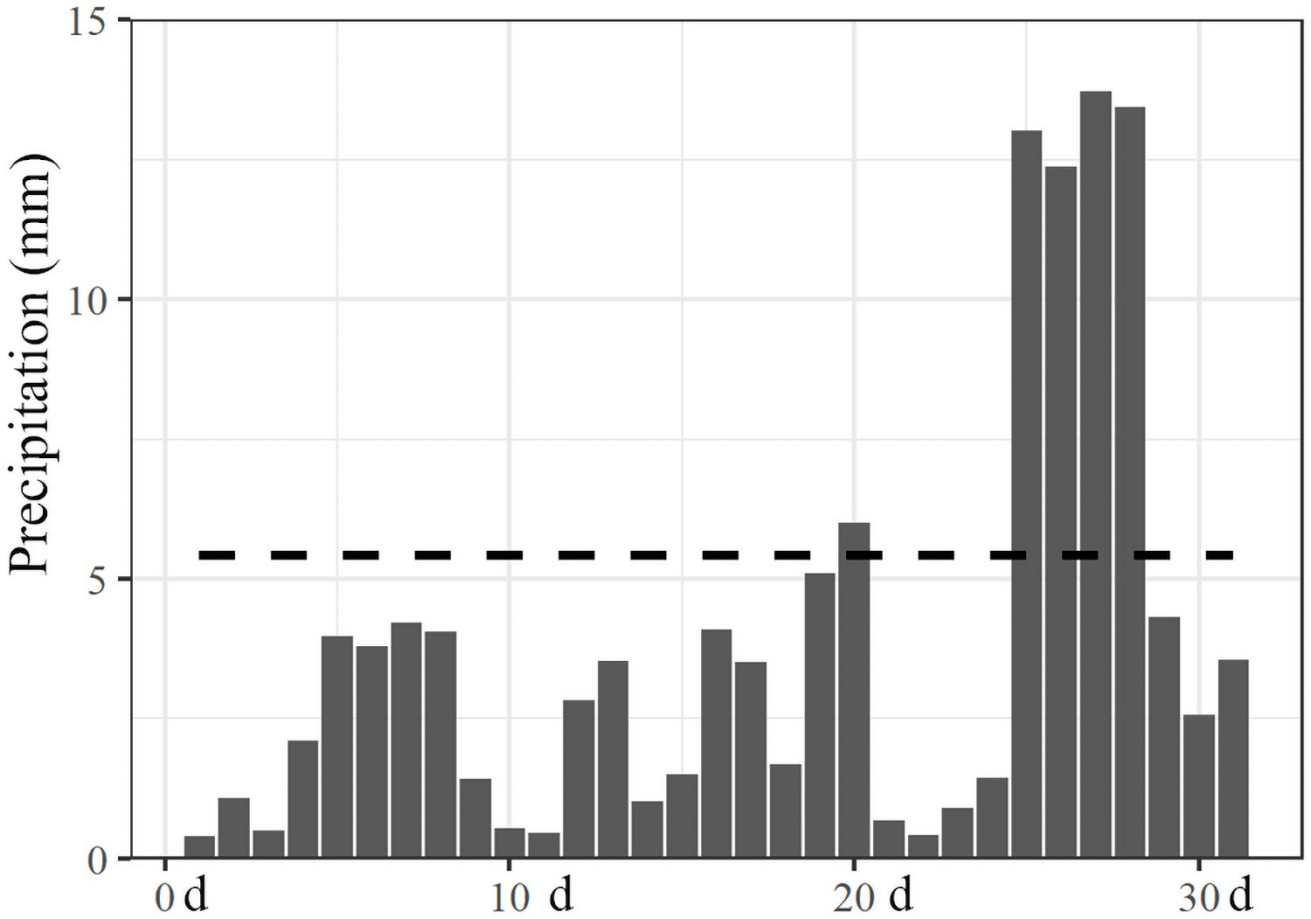
The daily precipitation of May in 2019. The dotted line is the average daily precipitation in May from 2000 to 2018.

After *C*. *oleifera* shooting period, the precipitation in Ceheng County between March and April in 2019 were comparable to those in the same time period between 2000 and 2018. In May, the precipitation (1182 mm) was lower than the normal range (1636 ± 383 mm), with a Pa value of 0.28, although this precipitation did no fall within the drought range on the monthly scale (Fig 2). However, further observation showed that the precipitation in May mainly concentrated in late May (Fig 3), which indicated that precipitation on the monthly scale might underestimate the drought condition in Ceheng in early summer. To keep consistent with the Pa index, we analyzed the precipitation data between April 25 and May 24 (totally, 30 d), and found that the precipitation in Ceheng in early summer of 2019 was 838 mm (the average in the considered years was 1387 mm), with a Pa value of 0.396. The precipitation during that period in 2019 was even lower than that in 2009 and in 2010 (these two years were drought years); that is, Ceheng suffered climate drought during that period. Nevertheless, the drought time lasted for one month, and the precipitation in June returned to normal.

### Anatomical structural characteristics of *C*. *oleifera* leaves in the low-hot valley

**Fig 4:**
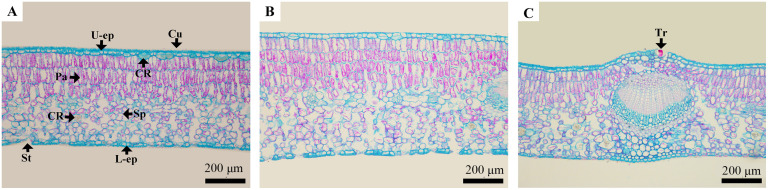
Structural characteristics of *Camellia oleifera* leaves from the low-hot valley using optical microscopy. A. C6 with three layers of palisade tissue leaves, ×100; B. C3 with four layers of palisade tissue leaves, ×100; and C. trichome on adaxial surface of the main vein of C15,×100. Notes: All bars: 200 μm. All pictures were taken under an optical microscope. All pictures were taken at 100 times. Cu: Cuticle; U-ep: Upper epidermis cells; Pa: Palisade tissue; Sp: Spongy tissue; L-ep: Lower epidermis cells; CR: Crystal; Tr: Trichome; and St: Stomata.

The leaf structure of *C*. *oleifera* consisted of cuticle, upper epidermis, palisade tissue, spongy tissue, lower epidermis and vein (Fig 4). Most upper epidermis contained monolayer cells with double-layer cells (multiple epidermis), and some of the second layer of upper epidermis cells contained crystals. The palisade tissue was mainly composed of three layers (Fig 4A), and some leaves had four layers (Fig 4B). The palisade cells in the upper layer were mainly columnar-shaped cells with a dense distribution, while the palisade cells in the lower layer contained short-type cells with a relatively sparse distribution. The palisade cells in each layer were arranged in an orderly manner, and the staining using the safranine O-fast green staining method was relatively uniform. The thickness of spongy tissue was slightly higher than that of palisade tissue, where crystal distribution (P/S ratio = 0.95) was observed. The adaxial surface of the leaf midrib was covered with thin hair (Fig 4C), while the upper and lower epidermis were hairless, with the upper epidermis thicker than the lower epidermis (U/L = 1.27).

### Differences in leaf structure of healthy individual plants

Palisade tissue and spongy tissue were the main components (90.34%) of the *C*. *oleifera* leaf structure. These tissues were associated with the growth and development of plants in terms of light energy fixation, and they also contained important indices of plant stress resistance. There were very significant differences in different leaf tissues of healthy *C*. *oleifera* plants from the low-hot valley (Tables 1 and 2).

**Table 1:** Leaf structure characteristics of healthy *C*. *oleifera* plants.

Plant number	LT (μm)	Palisade thickness (μm)	Spongy thickness (μm)	CTR	SR	P/S ratio
C2	422.3±20.71mn	163.7±8.48no	213.53±22.73ijk	38.86±2.94lmn	50.44±3.54abc	0.78±0.13klmn
C3	587.93±25.66b	236.9±15.06cd	295.17±34.15ab	40.4±3.62ijklmn	50.1±4.12abcd	0.82±0.14ijklmn
C4	480.59±16.07hij	199.91±13.18hijkl	225.32±6.64ghi	41.58±1.81ghiijklmn	46.92±2.05cdefghi	0.89±0.07fghijklmn
C5	511.97±15.49efg	194.93±14.99ijklm	266.31±10.54cde	38.06±2.36n	52.04±2.31a	0.73±0.08n
C6	475.57±12.01hij	199.12±11.28hijkl	225.41±17.41ghi	41.87±2.22ghiijklmn	47.38±3.25bcdefghi	0.89±0.09fghijklmn
C7	620.71±27.39a	265.43±14.38b	301.45±25.33a	42.84±2.99fghiijkl	48.51±2.36abcdef	0.89±0.1fghijklmn
C8	388.76±13.15o	155.38±11.94o	188.72±20.04kl	40.05±3.87jklmn	48.47±3.92abcdef	0.84±0.14hijklmn
C9	460.94±15.15ijk	185.15±15.82klm	229.53±24.11ghi	40.19±3.41iijklmn	49.75±4.47abcde	0.82±0.13hijklmn
C10	468.85±47.62ijk	194.64±26.52ijklm	225.37±35.86ghi	41.51±3.71ghiijklmn	47.93±4.66abcdefgh	0.88±0.15ghijklmn
C11	521.93±12.22e	208.16±15.57hij	263.47±20.94cde	39.89±2.91jklmn	50.48±3.77abc	0.8±0.1jklmn
C12	458.12±7.41ijkl	179.23±9.59mn	234.99±12.78fghi	39.14±2.34klmn	51.29±2.51ab	0.77±0.08lmn
C13	562.18±16.2bc	254.11±13.51bc	259.96±7.48def	45.19±1.7defg	46.26±1.29cdefghi	0.98±0.06cdefghi
C14	522.16±21.25e	203.45±15.84hijk	268.12±17.8cde	38.94±2.06lmn	51.36±2.86ab	0.76±0.07mn
C15	469.76±14.2ijk	194.15±13.74ijklm	229.59±25.2ghi	41.38±3.45ghiijklmn	48.83±4.75abcdef	0.86±0.19ghijklmn
C17	532.64±16.45de	228.9±25.24defg	252.44±28.71defg	42.97±4.41fghiijk	47.37±4.93bcdefghi	0.93±0.21efghijklm
C18	642.76±33a	297.83±17.94a	290.04±25.56abc	46.36±2.2cdef	45.07±2.3fghij	1.03±0.09cdefg
C19	467.16±48.45ijk	190.2±16.8jklm	229.62±42.55ghi	40.98±4.46hiijklmn	48.84±4.55abcdef	0.85±0.17hijklmn
C21	441.55±13.88klmn	204.71±17.42hij	185.15±19.18I	46.39±4.04cdef	41.9±3.74jklm	1.12±0.17bcd
C22	552.91±26.16cd	231.89±24.43def	272.47±31.38bcd	42.01±4.87ghiijklm	49.24±4.92abcdef	0.87±0.21ghijklmn
C23	479.24±8.57hij	211.06±16.2hi	220.72±18.25hi	44.03±3.15efghi	46.04±3.57defghi	0.96±0.13defghij
C24	476.94±15.88hij	215.22±13.15fgh	218.12±14.51hi	45.13±2.34defg	45.74±2.73efghij	0.99±0.11cdefgh
C25	489.32±11.17fghi	194.21±16.69ijklm	253.96±19.21drfg	39.72±3.68jklmn	51.88±3.34a	0.77±0.13klmn
C26	460.26±13.3ijkl	244±18.85cd	178.8±20.63i	53.04±4.22a	38.81±3.99m	1.39±0.24a
C27	456.92±17.06jkl	202.42±9.19hijk	216.82±20.92ij	44.36±2.62defgh	47.37±3.08bcdefghi	0.94±0.11efghijk
C29	524.43±45.05e	245.77±14.4cd	231.14±32.5ghi	47.03±2.93bcde	43.91±2.83hijk	1.08±0.13cde
C30	513.84±14.37ef	211.72±17.95ghi	247.05±17.57defg	41.18±2.97hiijklmn	48.08±3.16abcdefgh	0.86±0.11ghijklmn
C31	502.86±13.9efgh	217.83±14.61efgh	234.96±22.26fghi	43.34±2.94efghij	46.7±3.88cdefghi	0.94±0.14efghijkl
C33	433.31±35.08lmn	201.19±19.93hijkl	191.85±28.8jkl	46.55±4.52cdef	44.15±4.42ghijk	1.07±0.21cde
C35	450.49±6.56jklm	182.49±11.18lm	220.29±11.46hi	40.52±2.58hiijklmn	48.9±2.3abcdef	0.83±0.09hijklmn
C36	489.92±18.3fghi	206.61±12.61hij	241.66±17.89efgh	42.21±2.8ghiijklm	49.29±2.4abcdef	0.86±0.09ghijklmn
C37	482.05±12.25ghij	204.51±14.07hij	232.01±14.13ghi	42.41±2.5ghiijklm	48.15±3.1abcdefg	0.89±0.11fghijklm
C38	414.74±94.6no	206.77±40.06hij	166.27±43.99i	50.25±3.58ab	39.92±3.34lm	1.27±0.19ab
C39	471.23±19.26ijk	234.93±13.44de	190.82±14.92kl	49.87±2.51abc	40.46±1.9klm	1.24±0.12b
C40	515.35±48.9ef	245.06±10.76cd	225.85±48.89ghi	47.91±4.73bcd	43.4±5.85ijkl	1.14±0.28bc
C41	502.81±29.66efgh	233.05±10.12de	222.03±16.98hi	46.41±1.64cdef	44.13±1.3ghijk	1.05±0.07cdef
Mean	492.93	212.7	232.83	43.16	47.18	0.94
F value	34.14**	27.83**	14.35**	11.07**	7.78**	10.35**
C/V	12.03%	15.41%	16.83%	10.98%	10.08%	22.00%

Note: Different letters in the same column indicate significant differences at the 0.05 level;

** indicates that the difference is extremely significant (P<0.01).

**Table 2:** Leaf structure characteristics of healthy *C*. *oleifera* plants.

Plant number	Cu (μm)	Pa (μm)	U-ep (μm)	L-ep (μm)	Ratio of U/L
C2	8.33±0.82r	53.8±3.96m	22.7±3.83klm	22.57±2.24bcdefgh	1.01±0.14h
C3	17.33±0.99abc	82.05±6.49bcd	32.02±2.89bcd	26.77±2.18a	1.21±0.17defgh
C4	12.73±0.8p	73.82±6.93defg	25.94±5.32fghijk	23.14±2.06bcdefg	1.14±0.32efgh
C5	17.23±1.33bcd	66.31±6.09fghijkl	29.41±1.93cdefgh	23.26±2.73bcdefg	1.28±0.17bcdefg
C6	16.27±1.19defg	71.92±4.96efgh	33.75±6.31ab	24.95±2.18abcd	1.35±0.21bcdef
C7	13.86±0.89mo	65.39±7.81ghijkl	33.82±4.67ab	25.78±2.78ab	1.33±0.25bcdefg
C8	11.61±1.08q	61.11±7.34jklm	26.79±2.73efghijk	22.49±2.76cdefgh	1.2±0.16defgh
C9	14.94±1.09hijklm	62.74±9.89ijkl	24.97±3.33hijkl	19.69±3.02hijk	1.3±0.29bcdefg
C10	15.13±0.91hijkl	73.48±6.43defg	29.95±4.2bcdefg	21.57±2.44defghij	1.41±0.27bcde
C11	14.75±0.2ijklmn	77.27±5.99bcde	32.09±4.65bc	21.83±2.11cddefghij	1.48±0.25bc
C12	15.67±0.61ghij	58.86±9.44lm	25.52±4.94ghijk	23.01±2.94bcdefg	1.14±0.37efgh
C13	14.7±0.63jklm	65.18±7.83ghijkl	28±2.51cdefghij	26.56±3.7a	1.07±0.18gh
C14	14.38±0.31lmno	77.09±4.46bcde	30.37±5.98bcdef	21.84±1.67cddefghij	1.4±0.3bcde
C15	15.27±0.85ghijlk	61.11±7.29jklm	27.53±3.66dcefghij	19.96±2.5ghijk	1.4±0.27bcde
C17	13.82±1.16no	73.15±5.22defgh	26.35±3.49fghijk	24.16±4.35abcde	1.11±0.2fgh
C18	18.28±0.91a	104.89±7.5a	37.56±6.22a	22.34±3.69cdefgh	1.71±0.34a
C19	17.12±0.81bcd	73.12±9.22defgh	28.37±3.24cdefghij	19.08±2.04ijk	1.5±0.22ab
C21	12.64±1.4p	76.69±4.69bcde	31.25±5.39bcde	22.13±1.33cddefghi	1.42±0.26bcd
C22	17.81±1.39ab	70.82±7.62efghi	26.29±3.74fghijk	23.71±1.84abcdef	1.12±0.22fgh
C23	17.56±0.83abc	75.43±11.42cdef	29.29±3.1cdefghi	22.83±2.58bcdefgh	1.29±0.18bcdefg
C24	14.33±0.61lmno	64.64±6.65ghijkl	26.48±4.11fghijk	20.05±2.69ghijk	1.35±0.28bcdefg
C25	17±1.18bcd	69.63±12.12efghij	26.78±3.62efghijk	21.08±4.33efghijk	1.29±0.16bcdefg
C26	14.53±0.83klmn	71.19±11.14efghi	25.1±3.44hijkl	18.01±2.41k	1.42±0.27bcd
C27	13.45±1.54p	69.23±6.56efghij	24.11±2.65jklm	18.7±1.6jk	1.3±0.2bcdefg
C29	16.73±0.72cdef	73.56±6.23defg	29.21±4.51cdefghi	23.98±2.47abcdef	1.23±0.21cdefgh
C30	16.91±1.33bcde	81.46±7.98bcd	29.35±1.67cdefgh	24.04±2.53abcdef	1.23±0.15bcdefgh
C31	15.93±0.67efgh	82.87±6.79bc	27.93±2.05cdefghij	25.12±2.05abc	1.12±0.13fgh
C33	15.18±0.52hijkl	62.17±7.66ijklm	21.25±2.11lm	18.02±3.11k	1.21±0.21defgh
C35	15.58±0.88ghijk	64.18±3.83hijkl	28.09±1.87cdefghij	20.78±2.1fghijk	1.36±0.12bcdef
C36	13.86±0.64no	66.52±7.46fghijkl	24.69±2.7ijkl	21.09±2.5efghijk	1.19±0.2defgh
C37	13.9±0.97mno	75.89±6.42cde	26.55±2.84fghijk	21.77±2.22defghij	1.22±0.12cdefgh
C38	12.71±0.53p	59.82±13.09klm	20.57±5.03m	18.64±3.99jk	1.11±0.15fgh
C39	15.81±1.62fghi	68.6±8.33efghijk	28.71±2.88cdefghi	23.26±4.48bcdefg	1.29±0.35bcdefg
C40	17.57±0.87abc	71.11±8.52efghi	28.51±1.26cdefghij	22.89±2.91bcdefgh	1.26±0.15bcdefgh
C41	14.5±0.58lmno	84.88±11.45b	26.69±5.08fghijk	24.3±3.07abcde	1.12±0.29fgh
Mean	15.07	71.14	27.89	22.27	1.26
F value	41.61**	13.01**	7.77**	7.02**	3.69**
C/V	15.27%	16.68%	16.02%	18.17%	20.18%

Notes: Different letters in the same column indicate significant differences at the 0.05 level, and

** indicates that the difference is extremely significant (P<0.01).

**Fig 5:**
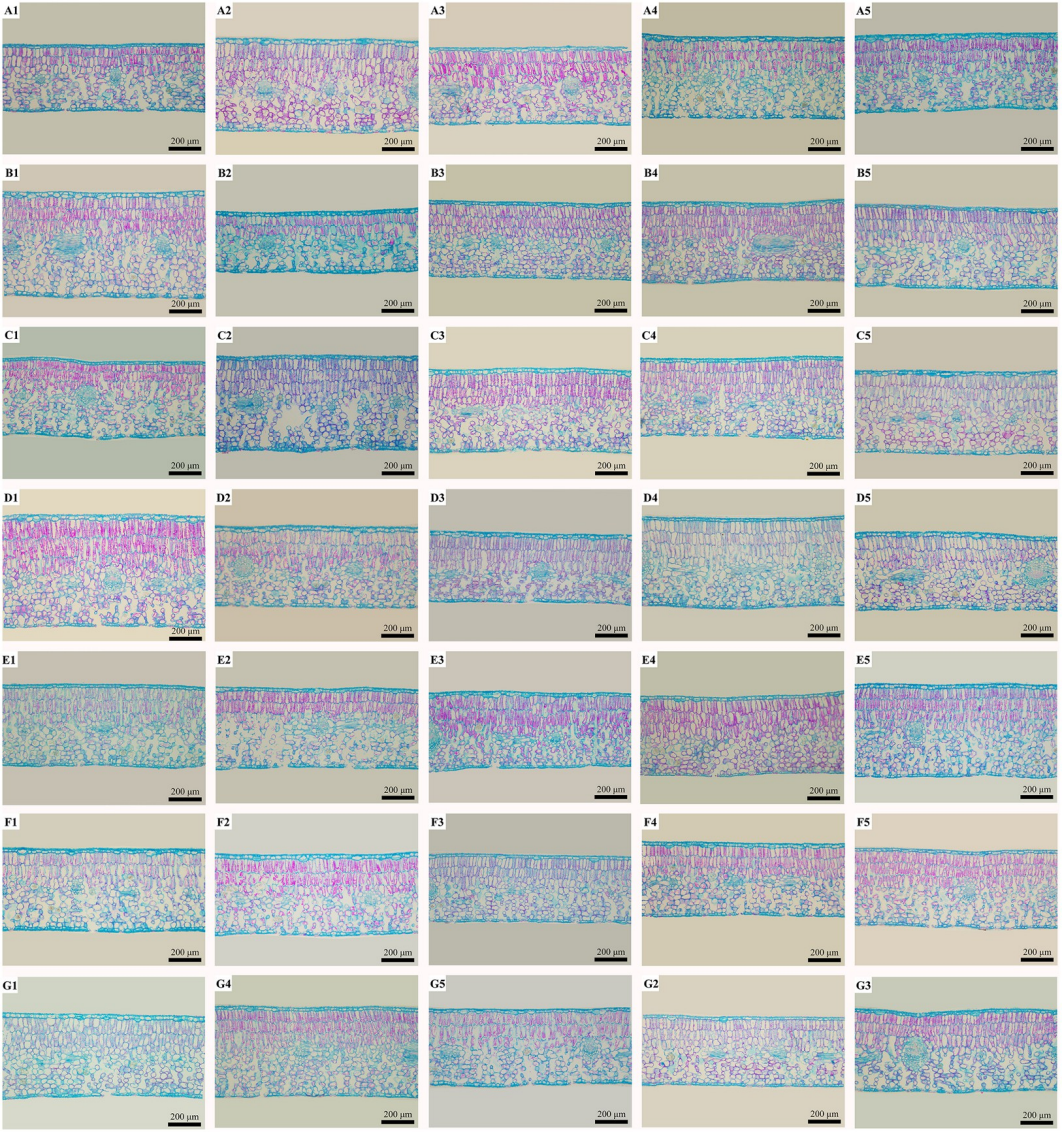
Leaf structure characteristics of 35 healthy *C*. *oleifera* plants using optical microscopy. A. Leaf structure characteristics of C2, C3, C4, C5 and C6, ×100; B. leaf structure characteristics of C7, C8, C9, C10 and C11, ×100; C. leaf structure characteristics of C12, C13, C14, C15 and C17, ×100; D. leaf structure characteristics of C18, C19, C21, C22 and C23, ×100; E. leaf structure characteristics of C24, C25, C26, C27 and C29, ×100; F. leaf structure characteristics of C30, C31, C33, C35 and C36, ×100; and G. leaf structure characteristics of C37, C38, C39, C40 and C41, ×100. Notes: All pictures were taken under an optical microscope; all photos were taken at a magnification of 100 times; bars: 200 μm.

The leaf thickness of C18 was the largest, reaching 642.77 μm. Its palisade tissue and upper epidermis were also the thickest, reaching 297.83 μm and 37.56 μm, respectively. At the same time, the U/L ratio was the greatest, measuring 1.68. The leaf thickness and palisade tissue of C8 were the smallest, measuring 388.76 and 388.76 μm, respectively. The U/L ratio of C2 was the smallest, measuring 1.01. The spongy tissue of C7 was thick, reaching 301.45 μm. The spongy tissue and upper epidermis of C38 were the thinnest, at 166.27 and 20.57 μm, respectively. The porosity of C5 was the largest, while the CTR and P/S ratio were the smallest, which were 38.08, 52.02 and 0.73 μm, respectively. The porosity of C26 was the smallest, while the CTR and P/S ratio were the largest, measuring 38.85, 53.01 and 1.36, respectively. In addition, the thickness of the lower epidermis was the thinnest, at 18 μm (Fig 5).

LT, palisade tissue, spongy thickness, upper epidermis, TFP and cuticle exhibited extremely significant correlations, while a significant correlation was found between lower epidermis and other tissues except for the cuticle, showing a coevolution of leaf tissue structure to adapt to the environment. There was a significant correlation between porosity and leaf thickness but no correlation between tightness and leaf thickness. There was no correlation between the P/S ratio and cuticle/upper epidermis thickness but a significant correlation between the P/S ratio and other tissues. The U/L ratio was extremely significantly correlated with cuticle thickness and TFP in addition to its extremely significant correlation with the upper and lower epidermis. Additionally, it was significantly correlated with palisade tissue thickness but was not correlated with other issues (Table 3).

**Table 3:** Correlation of leaf tissues and indices of healthy *C*. *oleifera* plants.

	LT	Palisade thickness	Sponge thickness	Cu	U-ep	Palisade 1	L-ep	CTR	SR	P/S ratio
Palisade thickness	.724**	1								
Spongy thickness	.819**	.230**	1							
Cu	.416**	.387**	.272**	1						
U-ep	.459**	.315**	.323**	.316**	1					
Palisade 1	.467**	.524**	.200**	.380**	.371**	1				
L-ep	.402**	.217**	.346**	.097	.303**	.184**	1			
CTR	-.092	.615**	-.596**	.100	-.070	.216**	-.156**	1		
SR	.185**	-.488**	.712**	-.049	-.010	-.218**	.113*	-.921**	1	
P/S ratio	-.161**	.539**	-.670**	.067	-.050	.196**	-.155**	.973**	-.966**	1
Ratio of U/L	.106	.130*	.016	.219**	.647**	.186**	-.512**	.072	-.110	.088

Note: Analysis using Pearson correlation,

* indicates that there is a significant correlation (P<0.05), and

** indicates that the difference is extremely significant (P<0.01).

According to the plasticity (P/I) analysis, LT (0.4), palisade tissue (0.48), spongy tissue (0.45), upper epidermis (0.45), lower epidermis (0.33), CTR (0.28), SR (0.25), U/L ratio (0.4) and P/S ratio (0.46) showed a certain plasticity, with the plasticity of palisade tissue being the highest, yet there was no significant difference in P/I between palisade tissue and other tissues. The P/I of leaf tissue might be one of the important characteristics for *C*. *oleifera* to adapt to the low-hot valley habitat.

### Evaluation of the leaf anatomical structure of healthy *C*. *oleifera* plants

In contrast to animals, forest trees are unable to avoid bad site conditions. The term “environment” can be used to describe the sum of the ambient conditional factors that affect the growth and development of animals and plants, while “site conditions” are specifically used for the environment in which forest trees grow. Plant leaves are assimilating organs, and the structural characteristics of their different internal tissues enable them to adapt to different site conditions.

In this study, a hierarchical cluster analysis was performed according to the average values of 11 leaf anatomical indices of 35 *C*. *oleifera* plants. The tissue structure indices were classified by Euclidean measurement, in which the cuticle thickness at 1.5 and the thickness of the lower epidermis were classified in the same category, inconsistent with the result that the cuticle and the lower epidermis were not correlated in the correlation analysis. Therefore, cosine was adopted for measurement in the cluster analysis of tissue structure, while Euclidean measurement was used for the cluster analysis of individual plants.

**Fig 6:**
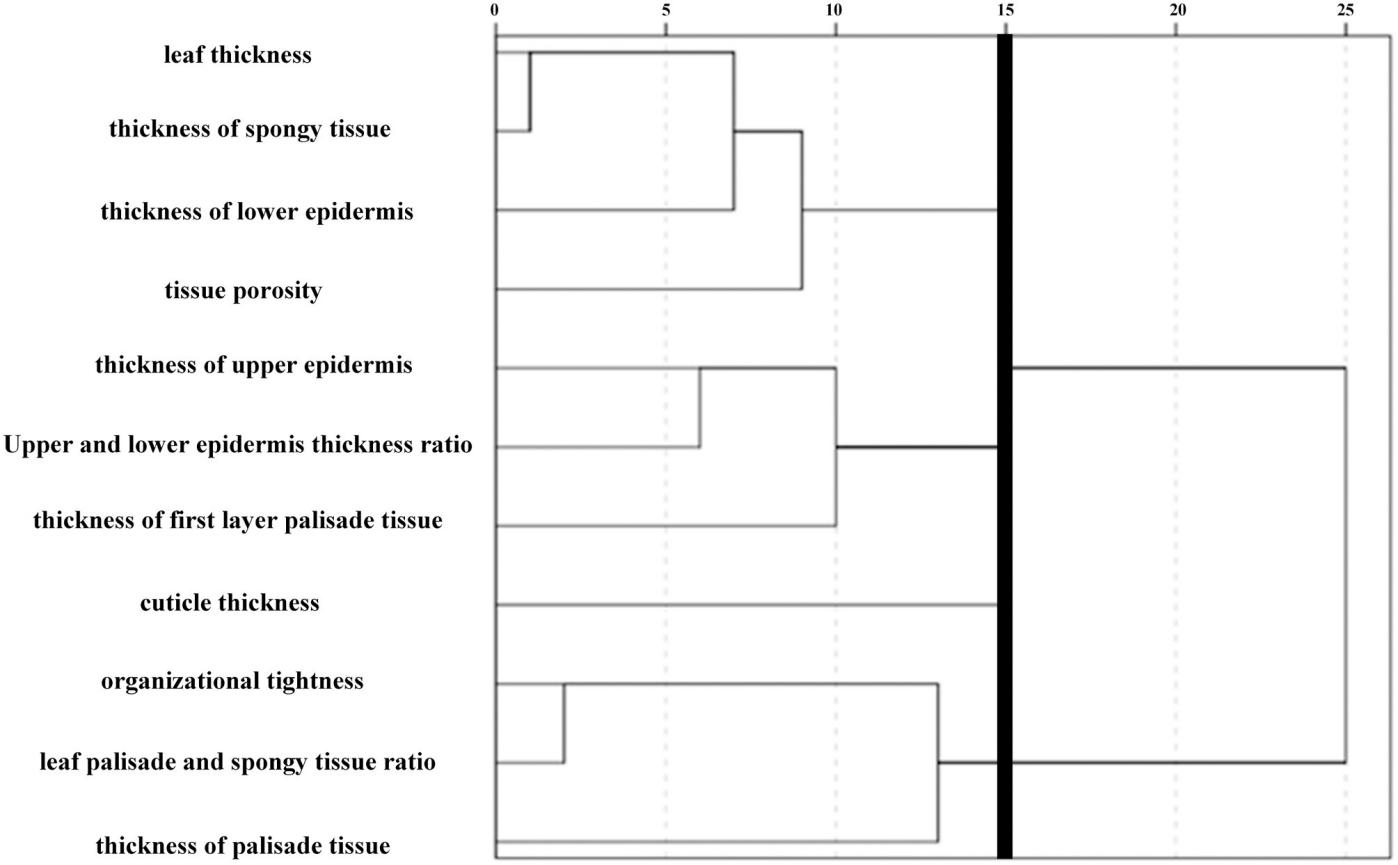
Cluster analysis of 11 leaf anatomical structures.

**Fig 7:**
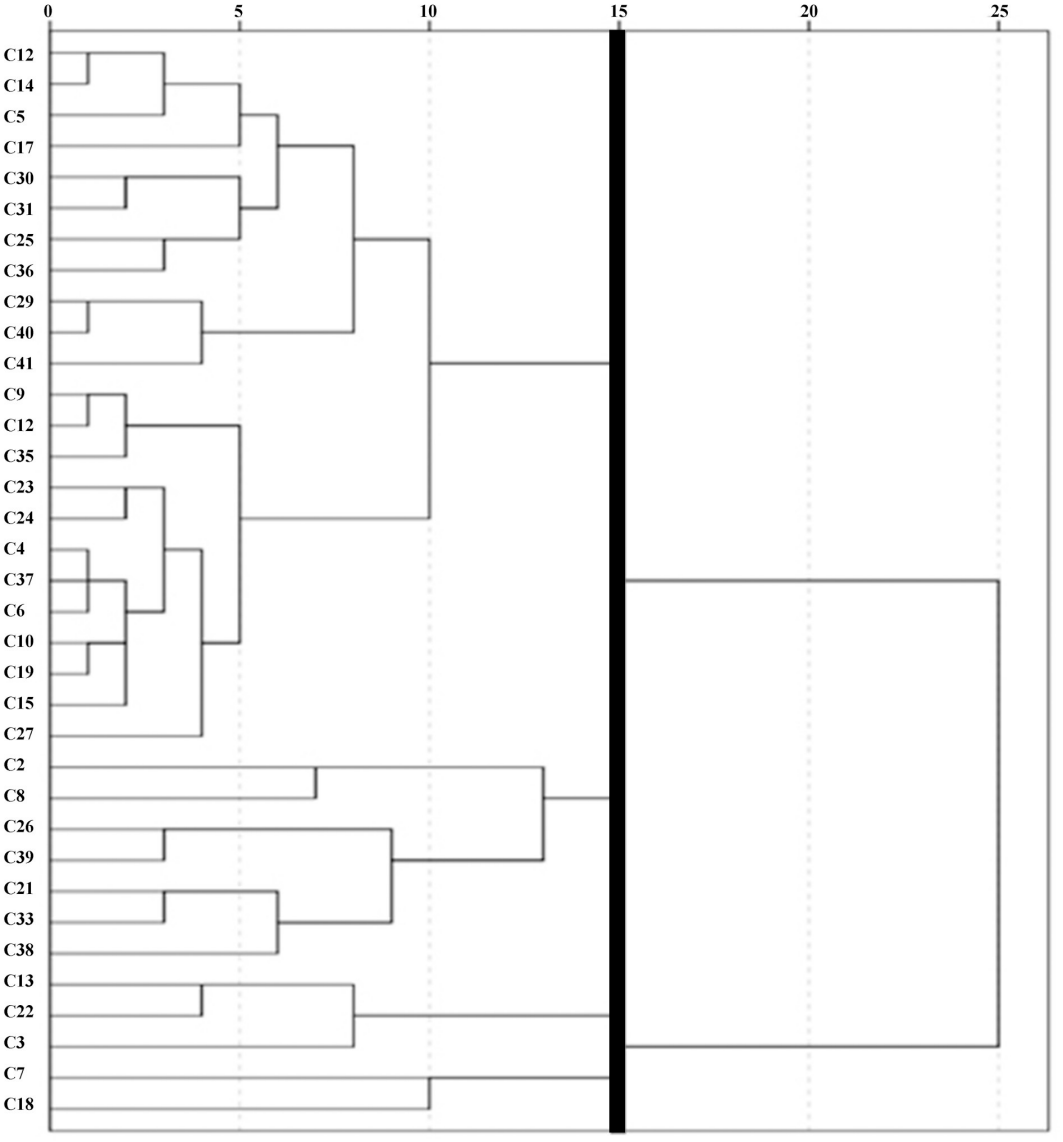
Cluster analysis of 35 healthy individual plants.

According to the clustering analytical results, 11 indices (a distance of 15) and 35 healthy individual plants (a distance of 15) were clustered into 4 categories, and the distance between these categories was relatively large (Figs 6 and 7). The first category of leaf structure indices included LT, spongy thickness, lower epidermis and SR; the second category included upper epidermis, U/L ratio and TFP; the third category included cuticle; and the fourth category included P/S ratio, CTR and palisade thickness.

The cluster analysis of healthy individual plants revealed that the first category contained the best individual plants, with 23 plants clustering in one category; the second category contained C2, C8, C21, C26, C33, C38, and C39; the third category contained C3, C13 and C22; and the fourth category contained only C7 and C18. The characteristics of each category are shown in Table 4.

**Table 4:** Structural characteristics of different leaf tissues.

Tissue type	1	2	3	4
LT (μm)	488.09	433.17	567.67	631.74
Palisade thickness (μm)	207.99	201.53	240.97	281.63
Spongy thickness (μm)	233.07	187.88	275.87	295.75
Cu (μm)	15.38	12.97	16.62	16.07
U-ep (μm)	27.79	25.2	28.77	35.69
Palisade 1 (μm)	71.64	64.77	72.68	85.14
L-ep (μm)	21.96	20.73	25.68	24.06
CTR	42.62	46.32	42.48	44.55
SR	47.75	43.54	48.58	46.84
Ratio of U/L	1.27	1.22	1.12	1.5
P/S ratio	0.88	1.07	0.87	0.95

According to the clustering results of tissue structure, the item with the greatest correlation index in the same category (Table 5) was selected. If the difference between the two items with the largest correlation index in the same category was not sufficiently large, the selection was made with reference to C/V (Tables 1 and 2). In the fourth category, the difference between the correlation indices of CTR (0.66) and the P/S ratio (0.62) was not sufficiently large, and the C/V of the P/S ratio was the largest among various indices, reaching 22.00%, and CTR was 10.98%. Therefore, the P/S ratio was selected as the evaluation index for the fourth category.

**Table 5:** Correlation classification and ordering of various indicators.

Category	Index	Correlation index	Ordering in the category
1	Spongy tissue	0.43	1
	LT	0.29	2
	SR	0.18	3
	L-ep	0.1	4
2	U-ep	0.28	1
	Ratio of U/L	0.23	2
	Palisade 1	0.09	3
3	Cu	1	1
4	CTR	0.66	1
	P/S	0.62	2
	Palisade tissue	0.33	3

Spongy tissue thickness, upper epidermis thickness, cuticle and P/S ratio were selected to evaluate and order the membership functions (Table 6). According to the ranking of the average membership degree of drought resistance and using the scoring difference of 0.2 to define one subrange (Table 5), the healthy individual plants of *C*. *oleifera* in the low-hot valley were divided and ranked into five categories: super strong > strong > medium > weak > none in terms of drought resistance. Only C18 had very strong drought resistance, and C3, C7 and C40 fell in the category of strong drought resistance. *Camellia oleifera* plants with medium drought resistance accounted for the majority, and included C29, C22, C6, C23, C30, C39, C11, C5, C13, C31, C14, C19, C26, C25, C10, C41, C17, C21, C15, C24, C35, C37 and C12. Plants with weak drought resistance included C36, C9, C4, C33, C27, C38 and C8. Only C2 had no drought resistance. In terms of distribution, the classification results have a normal distribution and therefore can be evaluated for significance (Table 6).

**Table 6:** Comprehensive evaluation of leaf tissues of 35 *C*. *oleifera* plants.

Individual plant	Spongy thickness	L-ep	P/S	Cu	Average membership degree	Scope of membership degree	Drought resistance
C18	0.92	1	0.45	1	0.84	>0.8	Super strong
C3	0.95	0.67	0.14	0.9	0.67	0.6~0.8	Strong
C7	1	0.78	0.24	0.56	0.65		
C40	0.44	0.47	0.62	0.93	0.62		
C29	0.48	0.51	0.53	0.84	0.59	0.4~0.6	Medium
C22	0.79	0.34	0.21	0.95	0.57		
C6	0.44	0.78	0.24	0.8	0.57		
C23	0.4	0.51	0.35	0.93	0.55		
C30	0.6	0.52	0.2	0.86	0.55		
C39	0.18	0.48	0.77	0.75	0.55		
C11	0.72	0.68	0.11	0.65	0.54		
C5	0.74	0.52	0	0.89	0.54		
C13	0.69	0.44	0.38	0.64	0.54		
C31	0.51	0.43	0.32	0.76	0.51		
C14	0.75	0.58	0.05	0.61	0.5		
C19	0.47	0.46	0.18	0.88	0.5		
C26	0.09	0.27	1	0.62	0.5		
C25	0.65	0.37	0.06	0.87	0.49		
C10	0.44	0.55	0.23	0.68	0.48		
C41	0.41	0.36	0.48	0.62	0.47		
C17	0.64	0.34	0.3	0.55	0.46		
C21	0.14	0.63	0.59	0.43	0.45		
C15	0.47	0.41	0.2	0.7	0.45		
C24	0.38	0.35	0.39	0.6	0.43		
C35	0.4	0.44	0.15	0.73	0.43		
C37	0.49	0.35	0.24	0.56	0.41		
C12	0.51	0.29	0.06	0.74	0.4		
C36	0.56	0.24	0.2	0.56	0.39	0.2~0.4	Weak
C9	0.47	0.26	0.14	0.66	0.38		
C4	0.44	0.32	0.24	0.44	0.36		
C33	0.19	0.04	0.52	0.69	0.36		
C27	0.37	0.21	0.32	0.51	0.35		
C38	0	0	0.82	0.44	0.32		
C8	0.17	0.37	0.17	0.33	0.26		
C2	0.35	0.13	0.08	0	0.14	<0.2	None

## Discussion

Plant leaves are the organs most susceptible to environmental regulation [34]. Their structural characteristics vary with environmental changes. This property is also known as the structural plasticity of leaves [9]. Plasticity is of great significance for immobile plants to adapt to abiotic stresses of different types and varying degrees [35,36]. Tree body mainly provides water for leaves, which receive more solar radiation compared with other plant organs [37]. In this study, we selected the leaves fully exposed to sunlight for investigation. The palisade tissue (0.48) and the spongy tissue (0.45) both displayed high plasticity, and these indices determined leaf thickness (r = 0.724 and 0.819, respectively). Under short-term drought stress, *Canarium album* and *Platanus orientalis* maintain moisture by increasing leaf thickness [3,38] while *Lycopersicon esculentum* decreases leaf thickness under long-term drought stress [39]. Under short-term drought stress, the decrease in the water content of leaves exhibits a certain pattern: The water content of non-functional leaves (lower leaves, morphologically) continuously decreases, whereas that of functional leaves (upper leaves) is maintained to keep biomass accumulation [40]. After rehydration, functional leaves maintain their strategy for drought. *Platanus orientalis* maintains moisture by increasing leaf thickness while *Platanus orientalis* exerts antioxidant protection by increasing non-enzymatics [38], which indicate the diversity of different species in dealing with even the same stress. In our study, the excellent individual plants underwent rehydration after short-term drought, and their drought resistance was assessed based on leaf anatomic structure. The plasticity of leaf thickness was 0.4. Presumably, the main reason is that the involved 35 plants were from the karst area where the habitat is complex, and therefore, their strategies to cope with drought stress differ. In the future, the physiological functions of the plants remain to be assessed.

The karst area has a high calcium environment. The Ca content of plants in this area is significantly higher than that in nonkarst areas. The Ca content in the aboveground part of plants is significantly higher than that in the belowground part [41]. Plants can mostly adapt to this site condition. *Lonicera confusa* leaves in karst areas store or remove excess calcium through glandular hairs and stomata under high Ca conditions [42]. When cultivated under high calcium conditions, a larger area of calcium oxalate crystals was formed beneath the cuticle of *Dracaena sanderiana* compared with low calcium culture [43]. At present, among the studies on the upper epidermis of *C*. *oleifera* leaves, the upper epidermis cells of different cultivars are uniformly composed of one layer of cells [22,44,45], while in the low-hot valley, a heterolayer phenomenon was observed in the upper epidermis cell layers. Calcium oxalate crystals were occasionally visible in the lower epidermis cells. An excessive supply of calcium and water loss contributes to the formation of calcium oxalate [46,47]. Calcium oxalate, closely related to the maintenance of ion balance in plants, is widely present in plants [48,49]. In addition, calcium oxalate crystals have a certain significance in taxonomy [50,51]. In the low-hot valley, a heterolayer phenomenon was observed in the upper epidermis in *C*. *oleifera* leaves, and crystals were distributed in the lower epidermis cells, which have not been discovered in *C*. *oleifera* leaves in other studies.

When plants are under drought conditions, maintaining photosynthesis is of essential importance for them to survive drought stress. In photosynthesis, CO_2_ diffuses into mesophyll cells (palisade tissue) through stomata, passes through multilayer cells and reaches chloroplasts in palisade tissue before being fixed there [52]. Palisade tissue is the main site where chlorophyll is distributed. Plants with short transport distances from stomata to chloroplasts (rather thin spongy tissue and thick palisade tissue—P/S ratio) may consume less energy and have a higher net photosynthetic rate. Therefore, there is a significant positive correlation between the net photosynthetic rate of *C*. *oleifera* and the P/S ratio, yet no significant correlation between the net photosynthetic rate and palisade tissue [53,54]. For *C*. *oleifera* in the most suitable cultivation area, its P/S ratio ranged from 0.6 to 0.8 [54], lower than that (P/S ratio = 0.94) in healthy *C*. *oleifera* plants in the low-hot valley. These results may be responsible for the higher net photosynthetic rate of *C*. *oleifera* leaves in the low-hot valley than in other regions, making it easier for *C*. *oleifera* in the low-hot valley to survive the drought period.

The cuticle, covering the upper epidermis, serves as the boundary between the plant leaves and the environment, protecting plants from biotic or abiotic stresses, and it is the main barrier to limit nonstomatal water loss, thereby promoting the survival of plants under drought conditions [55–58]. *Arabidopsis thaliana*, whose cutin synthesis was impeded, displayed intolerance to drought at its seedling stage, and its leaves showed signs of necrosis [59]. Research showed that the synthesis of the main structural components of the cuticle was induced by drought [60]. A thick cuticle and upper epidermis are also characteristics of plants in arid areas [61]. These descriptions might explain why the cuticle was one drought resistance evaluation factor according to this study.

The carbon starvation hypothesis for plant mortality due to drought [62] holds that under drought conditions, the stomata of plants are closed by hydraulic pressure to prevent water loss, which diminishes the absorption of carbon. However, the respiratory demand caused by high temperature accompanying drought aggravates the metabolic demand for carbon and causes plants to die of carbon starvation. To cope with drought conditions, plants often reduce aboveground biomass and increase belowground biomass to increase the probability of water availability. Therefore, the root system is well-developed. Only with this adaptation, however, CO_2_ required for photosynthesis is still insufficient. Through carbon starvation culture, calcium oxalate crystals in *Amaranthus hybridus* and *Colobantus quitensis* leaves significantly decreased. The degradation of crystals accompanied by the enhancement in oxalate oxidase activity might be attributable to the release of CO_2_ from the decomposition of calcium oxalate crystals to function as the substrate of photosynthesis to maintain photosynthesis [63–65]. In relevant research on the structure of *C*. *oleifera* leaves, the distribution of crystals was observed only in a few varieties [66], and crystals were only found in the leaf margin and parenchymatous tissue under the main vein [22]. In the *C*. *oleifera* leaves from the low-hot valley, crystals widely existed in the spongy tissue, which might be because the soil in the karst area contained a large amount of Ca. The content of Ca in the *C*. *oleifera* leaves was the highest among the contents of minerals in the region [67], which was an indication that plants in the karst region adapted to the environment, manifested in the storage of calcium oxalate crystals in *C*. *oleifera* from the low-hot valley. Greater leaf thickness is conducive to the storage of calcium oxalate crystals, while the determination coefficient of spongy tissue thickness and leaf was the largest (r2 = 0.671), greater than that of palisade tissue (r^2^ = 0.524). On the other hand, the conduction of CO_2_ in leaves is divided into stomatal conductance and mesophyll conductance. Drought reduces mesophyll conductance and causes the stomata to diminish in size [68–70]. Thicker spongy tissue may increase CO_2_ binding sites [3,71], promote the diffusion of gaseous CO_2_, and alleviate the reduction in CO_2_ caused by the closure of the stomata. However, in most studies on the effect of drought stress on leaf structure, spongy tissue is inversely proportional to the degree of stress. Contrary to this study, this may be because we used yield as the indicator to select healthy individual plants. The environmental stress on the selected healthy individual plants did not reach the extent to which it reduced the thickness of spongy tissue.

The phenomenon of soil erosion is serious in the low-hot valley, which results in the formation of a complex habitat. A single evaluation index cannot reflect the adaptability of *C*. *oleifera* to different habitats. Therefore, it is one-sided. Multiple indices jointly adopted for evaluation can effectively evaluate the interaction between the indices. There have been quite a few studies on the evaluation of plant stress resistance using the membership function method. Guo et al. [72] evaluated the cold resistance of different varieties of *Prunus persica* through the membership function, and the evaluation results were basically consistent with the field survey results. Zou [73] evaluated the drought resistance of *Gossypium hirsutum* varieties using physiological indices and the membership function and carried out evaluation verification of replanting under drought conditions. The results were consistent with the evaluation by the membership function, which has been widely adopted for the evaluation of plant stress resistance. In this study, we selected the cuticle, upper epidermis, spongy tissue and P/S ratio for a comprehensive evaluation and discussed these indices in the previous section. However, further study is still needed to verify these physiological indices and results.

In this study, we found that the leaf anatomical structure of *C*. *oleifera* from the low-hot valley was significantly different from that in other distribution areas. The *C*. *oleifera* leaf from the low-hot valley had a higher P/S ratio, and a distinct distribution of calcium oxalate crystals was observed in the mesophyll, which reflects the adaptability of the *C*. *oleifera* leaf structure to this area. Through the evaluation of the membership function, we screened four healthy *C*. *oleifera* plants with desirable drought resistance, namely, C18, C3, C7 and C40. Although the drought resistance of C39 and C26 was medium, they had a higher P/S ratio, making them promising for cultivating high-yield *C*. *oleifera* plants. These findings will provide a theoretical basis for the selection of breeding materials, will promote purposeful breeding of healthy cultivars and will decrease breeding costs.

## Supporting information

S1 Table Yields per unit crown width of 45 *Camellia Oleifera* plants. (DOCX)

S2 Table Original of the excellent *Camellia Oleifera* plants. (DOCX)
