# Supplementary material for: A comparative study on the leaf anatomical structure of Camellia oleifera in a low-hot valley area in Guizhou Province, China
Source: PLoS One. 2022 Jan 20;17(1):e0262509. doi: 10.1371/journal.pone.0262509 (PMC8775352; doi:10.1371/journal.pone.0262509)
Supplement: S1 Table — (DOCX) [file pone.0262509.s001.docx]

**S1 Table. Yields per unit crown width of 45 *Camellia Oleifera* plants**

| Serial No. | Yield per unit crown width (kg/m^2^) |  | Serial No. | Yield per unit crown width (kg/m^2^) |  |
| --- | --- | --- | --- | --- | --- |
| C-1 | 2.45±0.19 |  | C-24 | 4±1.15 |  |
| C-2 | 1.89±0.17 |  | C-25 | 3.32±0.79 |  |
| C-3 | 2.01±0.58 |  | C-26 | 3.41±0.25 |  |
| C-4 | 4.02±1.3 |  | C-27 | 2.56±0.38 |  |
| C-5 | 3.01±0.74 |  | C-28 | 2.03±0.30 |  |
| C-6 | 5.96±1.05 |  | C-29 | 2.91±1.03 |  |
| C-7 | 4.22±0.31 |  | C-30 | 5.77±0.44 |  |
| C-8 | 2.09±1.01 |  | C-31 | 5.26±1.14 |  |
| C-9 | 2.45±0.91 |  | C-32 | 4.04±0.89 |  |
| C-10 | 1.93±0.17 |  | C-33 | 4.28±0.84 |  |
| C-11 | 5.12±0.89 |  | C-34 | 2.41±0.95 |  |
| C-12 | 4.17±0.8 |  | C-35 | 1.94±0.67 |  |
| C-13 | 4.19±0.58 |  | C-36 | 5.03±0.91 |  |
| C-14 | 3.54±0.71 |  | C-37 | 2.9±0.15 |  |
| C-15 | 3.52±1.02 |  | C-38 | 1.79±0.37 |  |
| C-16 | 5.84±0.85 |  | C-39 | 2.28±0.4 |  |
| C-17 | 4.65±0.45 |  | C-40 | 4.78±0.37 |  |
| C-18 | 3.01±0.31 |  | C-41 | 1.78±0.34 |  |
| C-19 | 2.22±0.81 |  | C-42 | 1.77±0.19 |  |
| C-20 | 2.65±0.68 |  | C-43 | 1.73±0.93 |  |
| C-21 | 5.09±0.47 |  | C-44 | 1.56±0.19 |  |
| C-22 | 2.41±0.41 |  | C-45 | 1.21±0.28 |  |
| C-23 | 3.05±0.69 |  |  |  |  |
