# Supplementary material for: A comparative study on the leaf anatomical structure of Camellia oleifera in a low-hot valley area in Guizhou Province, China
Source: PLoS One. 2022 Jan 20;17(1):e0262509. doi: 10.1371/journal.pone.0262509 (PMC8775352; doi:10.1371/journal.pone.0262509)
Supplement: S2 Table — (DOCX) [file pone.0262509.s002.docx]

S2 Table. Original of the excellent *Camellia Oleifera* plants

| Origin | Serial No. | Origin | Serial No. |
| --- | --- | --- | --- |
| Biyou | C1 | Yangba | C30 |
|  | C2 |  | C31 |
|  | C3 |  | C32 |
|  | C4 |  | C33 |
|  | C5 |  | C34 |
|  | C6 |  | C35 |
|  | C7 |  | C36 |
|  | C8 |  | C37 |
|  | C9 |  | C41 |
|  | C10 |  | C42 |
|  | C11 |  | C43 |
|  | C12 |  |  |
|  | C13 |  |  |
|  | C14 |  |  |
|  | C15 |  |  |
|  | C16 |  |  |
|  | C17 |  |  |
|  | C18 |  |  |
|  | C19 |  |  |
|  | C20 |  |  |
|  | C21 |  |  |
|  | C22 |  |  |
|  | C23 |  |  |
|  | C24 |  |  |
|  | C25 |  |  |
|  | C26 |  |  |
|  | C27 |  |  |
|  | C28 |  |  |
|  | C29 |  |  |
|  | C38 |  |  |
|  | C39 |  |  |
|  | C40 |  |  |
|  | C44 |  |  |
|  | C45 |  |  |
